# Associations between urinary phytoestrogen mixed metabolites and osteoarthritis risk

**DOI:** 10.1371/journal.pone.0313675

**Published:** 2024-11-14

**Authors:** Dichao Huang, Hua Wang, Shuguang Wang, Tianming Yu, Long Zhou

**Affiliations:** 1 Department of Orthopedics, Ningbo No 6 Hospital, Ningbo, Zhejiang, China; 2 Department of Medical Imaging, Ningbo No 6 Hospital, Ningbo, Zhejiang, China; University of Vermont College of Medicine, UNITED STATES OF AMERICA

## Abstract

**Background:**

This study aims to explore the relationship between urinary phytoestrogen mixed metabolites and the risk of osteoarthritis (OA).

**Methods:**

Using data from the National Health and Nutrition Examination Survey (NHANES), a Weighted Quantile Sum (WQS) regression analysis was conducted to determine the dominant metabolites. Additionally, a Bayesian kernel machine regression (BKMR) model was utilized to explore the combined effects of phytoestrogen mixed metabolites on OA.

**Results:**

Compared to the lowest quartile group, the highest quartile group of Enterodiol showed a 46% increased risk of OA (OR = 1.46, 95% CI: 1.09–1.96), while the highest quartile group of Enterlactone showed a 30% decreased risk of OA (OR = 0.70, 95% CI: 0.52–0.96). The WQS regression model analysis revealed a positive relationship between urinary phytoestrogen mixed metabolites and OA risk, with Enterodiol found to have the highest weight in this association. The BKMR model indicated that the association between urinary phytoestrogens and OA increased with concentration but did not reach statistical significance. The univariate exposure-response function demonstrated a positive association between Enterodiol and OA.

**Conclusions:**

There is a positive relationship between urinary phytoestrogen mixed metabolites and OA, with Enterodiol being an important factor influencing OA risk.

## 1 Introduction

Osteoarthritis (OA) is a prevalent degenerative joint disease characterized by the progressive destruction of cartilage, remodeling of subchondral bone, and inflammation of the synovium. It represents the most common form of arthritis and is recognized as the primary cause of both chronic pain and long-term disability among adults [1–3]. The prevalence of OA is escalating as a result of an aging population and rising obesity rates, thus posing a formidable global public health challenge. Research indicates that in 2020, the number of individuals worldwide suffering from OA amounted to approximately 595 million, reflecting a staggering 132.2% surge in cases relative to 1990. Projections indicate that the global prevalence of OA will escalate to 1.102 billion individuals by the year 2050 [4].

The pathogenesis of OA remains incompletely understood, with various factors like age, obesity, hormonal changes, joint damage, and genetics influencing its development [5–7]. Of particular interest is the role of estrogen in OA, as an increasing body of evidence suggests a close relationship between estrogen levels and the risk of OA. A meta-analysis revealed that the incidence of OA in women over 50 years old exceeded that of men, indicating a potentially higher susceptibility to OA in postmenopausal women [8]. A cross-sectional study conducted in Italy involving 42,462 adult women demonstrated an elevated risk of OA in women with natural or surgical menopause [9]. Similarly, an epidemiological survey in Spain found that the risk of hand OA in women reached its peak around menopause, presenting a more than 3.5-fold increase in the incidence of hand OA in women aged 50–60 years compared to men of similar age [10]. Furthermore, animal experiments have shown that the estrogen levels of rats in the experimental group decreased, leading to increased hardness of subchondral bone remodeling. This alteration in biomechanical conditions of the joint resulted in exacerbated damage to articular cartilage and subsequently the development of OA [11, 12].

Hormone replacement therapy (HRT) has been found to demonstrate a protective effect on articular cartilage in postmenopausal women. However, it is important to note that HRT can also lead to various complications such as gynecological tumors, coronary heart disease, and stroke [13, 14], which has limited its clinical application. Phytoestrogen, a group of plant compounds bearing chemical similarity to mammalian estrogen, exhibits estrogen-like or antiestrogen-like effects through interaction with estrogen receptors [15]. The primary constituents of phytoestrogen include isoflavones and lignans. Isoflavones are predominantly found in beans and soy products, while lignans are primarily sourced from seed oil and whole wheat grains [16].

Given the diversity in food sources and biochemical structures of each phytoestrogen, it is crucial to examine the comprehensive effects of phytoestrogen in parallel with the relationship between individual phytoestrogens and disease outcomes. Preclinical investigations have demonstrated that equol can effectively delay cartilage degradation in a knee arthritis rat model by inhibiting the NF-κB signaling pathway and chondrocyte apoptosis [17]. Moreover, in vitro cell culture experiments have indicated that daidzein, a constituent of soy isoflavones, may potentially play a beneficial role in maintaining the phenotype of human chondrocytes and facilitating extracellular matrix formation [18]. Although numerous studies have focused on animal experimentation involving the relationship between phytoestrogen and OA, primarily investigating a single chemical, there is currently a lack of epidemiological investigations exploring the association between urinary phytoestrogens, mixed metabolites, and the risk of OA.

The aim of this study is to utilize data from the National Health and Nutrition Examination Survey (NHANES) spanning the years 2003 to 2010, in order to examine the potential impact of phytoestrogen mixed metabolites on the risk of OA.

## 2 Material and methods

### 2.1 Study population

The data utilized for this study were obtained from the NHANES survey, a regular survey conducted since 1960 by the Centers for Disease Control and Prevention. The purpose of this survey is to evaluate the health and nutritional status of both adults and children in the United States. All surveys conducted were subject to review and approval by the Ethics Review Board of the National Center for Health Statistics Research, and all participants provided written informed consent. In this particular study, publicly available data from participants recruited between 2003 and 2010 were analyzed. A total of 36,994 patients with OA were included based on self-reported information. Exclusions were made for 27,888 individuals with missing data on the six phytoestrogen measurements and four individuals with missing data on urine creatinine. Additionally, 3,672 individuals under the age of 20 were further excluded. Consequently, a total of 5,434 participants were enrolled in the study, including 582 patients diagnosed with OA. All methods were performed in accordance with the relevant guidelines and regulations.

### 2.2 Definition of OA

OA is diagnosed in the NHANES by a qualified medical professional, with relevant information being gathered through a questionnaire. Specifically, participants aged 20 years or older are required to respond to two specific inquiries related to arthritis. The first question posed to them is, "Have you ever been informed by a physician or any other healthcare professional that you have arthritis?" Subsequently, those who responded affirmatively were further queried with the subsequent question, "Which specific type of arthritis do you have?" It is important to note that only individuals who specified "OA" as their response were considered for inclusion in the study.

### 2.3 Measurement of urinary phytoestrogen

Urine specimens were collected at a mobile examination center and stored at -20°C. The concentrations of urine isoflavones (Daidzein, genistein, equol, and Q-desmethylangolensin), as well as lignans (enterodiol and enterolactone), were determined using high-performance liquid chromatography-tandem mass spectrometry. In instances where the measurement fell below the lowest limit of detection, the value was imputed by dividing the limit of detection by the square root of 2. Urinary phytoestrogen concentrations were corrected using urinary creatinine [19].

### 2.4 Covariates

Participant characteristics were extracted from the NHANES database, including age (in years), sex (male or female), race/ethnicity (Mexican American, other Hispanic, non-Hispanic white, non-Hispanic black, or others), marital status (married/cohabited, widowed/divorced/separated, or never married), alcohol use status (ever or never), smoking (never, past, present), body mass index (BMI), household income to poverty ratio (PIR), and serum cotinine concentration. Smoking and alcohol consumption were self-reported, while serum cotinine (creatinine) levels were measured using isotope dilution-high-performance liquid chromatography/atmospheric pressure chemical ionization tandem mass spectrometry. In cases where the result fell below the minimum limit of detection, the creatinine concentration was calculated as the square root of half the limit of detection.

### 2.5 Statistical analysis

The Kolmogorov-Smirnov (K-S) method was employed to assess the normality of the measurement data. For normally distributed data, the mean ± standard deviation (SD) was used for description, and an independent sample t-test was conducted to compare between groups. Non-normal data were described using the median and interquartile range, denoted as M (P25, P75), and the Wilcoxon rank-sum test was employed to compare between groups. Enumeration data were described in terms of the number of cases (constituent ratio), and comparison between groups was carried out using the Pearson test. In order to achieve an approximate normal distribution of phytoestrogen concentrations after correcting for urinary creatinine, a natural log transformation (ln transformation) was applied.

Phytoestrogen concentrations were categorized into quartiles. A generalized linear regression model was employed to analyze the relationship between individual urinary phytoestrogen metabolites—both ln-transformed and quartile-transformed—and the risk of OA, yielding odds ratios (ORs) and their corresponding 95% confidence intervals (CIs). To further identify the predominant metabolites, the Weighted Quantile Sum (WQS) regression model was used to examine the impact of mixed urinary phytoestrogen metabolites on OA. The WQS regression model is a statistical approach that categorizes exposures into ordered variables, assigns weighted scores based on their empirical weights within the mixture, and conducts linear or logistic regression analysis for continuous or binary outcomes, respectively. This method has been widely applied to investigate the effects of multiple mixture exposures on disease outcomes [20, 21]. In the WQS model, we utilized a binomial distribution as the link function. To calculate the CIs and P-values, we used the results from the quartile model and 5,000 bootstrap samples. Additionally, a Bayesian kernel machine regression (BKMR) model was applied to explore the combined effects of mixed phytoestrogen metabolites on OA and to investigate potential interactions among these metabolites. The BKMR model is capable of identifying non-linear and non-additive associations between exposure and outcome, as well as selecting important components of the mixture through variable selection. Its hierarchical variable selection approach addresses the issue of multicollinearity when individual components cannot be distinguished [22]. We fitted the final model with a Markov Chain Monte Carlo (MCMC) sampler for 50,000 iterations, obtaining the posterior inclusion probabilities (PIPs) for each phytoestrogen metabolite and estimates for the exposure-outcome functions.

Statistical analysis was performed using R 3.4.3 software, and a two-sided P-value of less than 0.05 was considered statistically significant.

### 2.6 Ethics statement

This study was based on a publicly available databases and does not require ethical approval. All of the participants or their legal representatives provided written informed consent during recruitment.

## 3 Results

### 3.1 The baseline characteristics of the study population were marked with the label and order of the chart and table

This study included a total of 5434 subjects. The baseline characteristics can be found in S1 Table. Of these subjects, 582 (10.7%) were diagnosed with OA, while the remaining 4852 (89.3%) were non-OA patients. Statistical analysis revealed significant differences (P<0.001) in age, gender, race, marital status, smoking status, drinking status, and body mass index between OA and non-OA patients.

### 3.2 Generalized linear regression model

The association between urinary phytoestrogen and the risk of OA was analyzed using the generalized linear regression model. The results, presented in S2 Table, indicate that compared to the lowest quartile (Q1) group, the highest quartile (Q4) group of Enterodiol exhibited a 46% increased risk of OA (OR = 1.46, 95%CI: 1.09–1.96). Conversely, both the third and highest quartile groups of Enterolactone demonstrated a reduction in OA risk, with a 35% decrease (OR = 0.65, 95%CI: 0.48–0.88) and 30% decrease (OR = 0.70, 95%CI: 0.52–0.96), respectively. Moreover, the analysis of logarithmically transformed phytoestrogen concentrations revealed that the risk of OA increased by 14% for each unit increase in Enterodiol and Enterolactone concentrations (OR = 1.14, 95%CI: 1.04–1.25), while Enterodiol and Enterolactone concentrations were associated with a 14% reduction (OR = 0.86, 95%CI: 0.78–0.95) in OA risk.

### 3.3 Weighted quantiles and regression (WQS regression)

**Fig 1:**
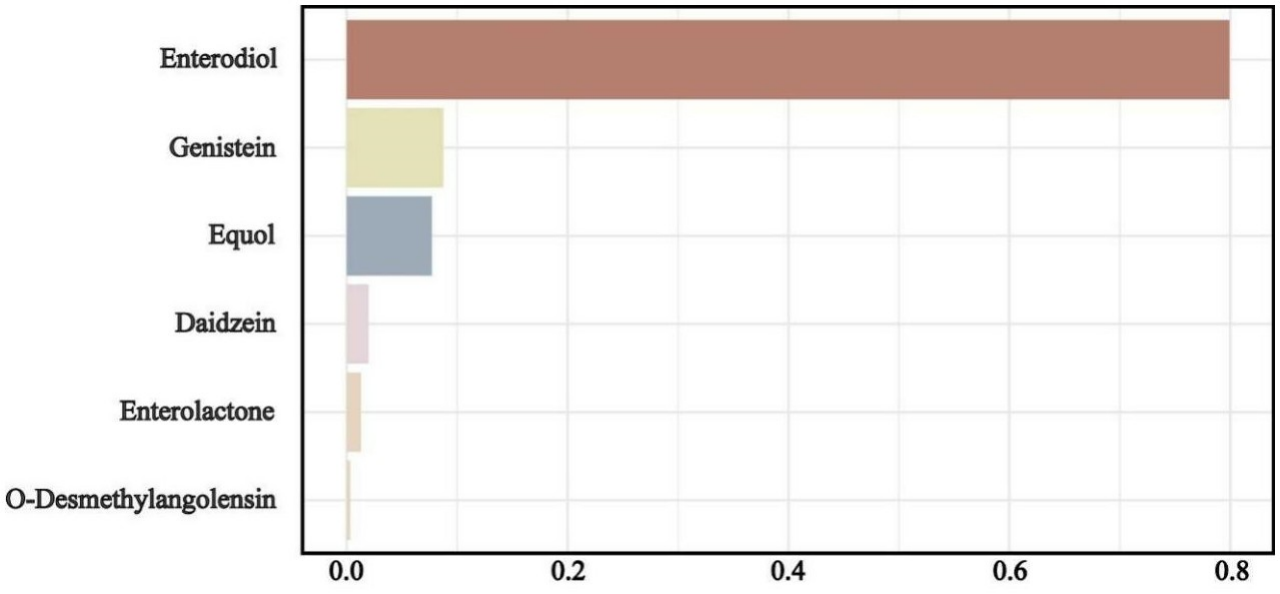
WQS model regression index weights for osteoarthritis.

The WQS regression index exhibited a significant relationship with the risk of OA (OR = 1.18, 95%CI: 1.02–1.35), as depicted in S3 Table. Furthermore, Fig 1 illustrates that Enterodiol, Genistein, and Equol had relatively elevated weights of 0.800, 0.098, and 0.096, correspondingly, in the WQS model.

### 3.4 Bayesian Kernel Machine Regression (BKMR)

**Fig 2:**
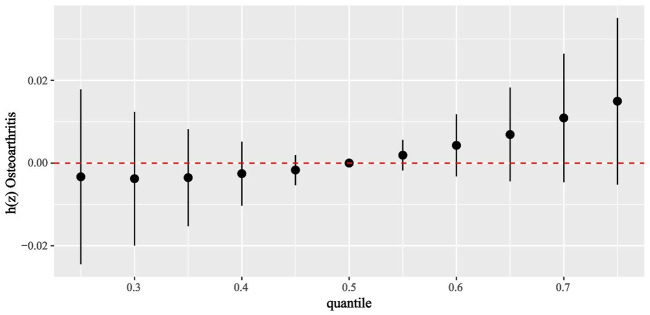
Associations of the urinary phytoestrogens with OA risk estimated by Bayesian Kernel Machine Regression (BKMR). This plot showed the estimated difference in OA risk and 95% confidence interval when all phytoestrogens concentrations were held at particular percentiles compared to their medians. Models were adjusted for sex, age, race, education, family income-to-poverty ratio, marital status, body mass index, smoking status, drinking alcohol status, and serum cotinine.

The concentration of phytoestrogens in urine was transformed using natural logarithm and considered as a continuous variable. The BKMR model was employed to examine the combined impact of urinary phytoestrogen on OA. Fig 2 provides an overview of the collective influence of six urinary phytoestrogen metabolites on the likelihood of developing OA. While no statistically significant differences were observed for high concentrations of individual metabolites when compared to their 50th percentile counterparts, there was an upward trend in the overall effect of metabolic combinations in the 60th percentile and above with respect to OA risk.

**Fig 3:**
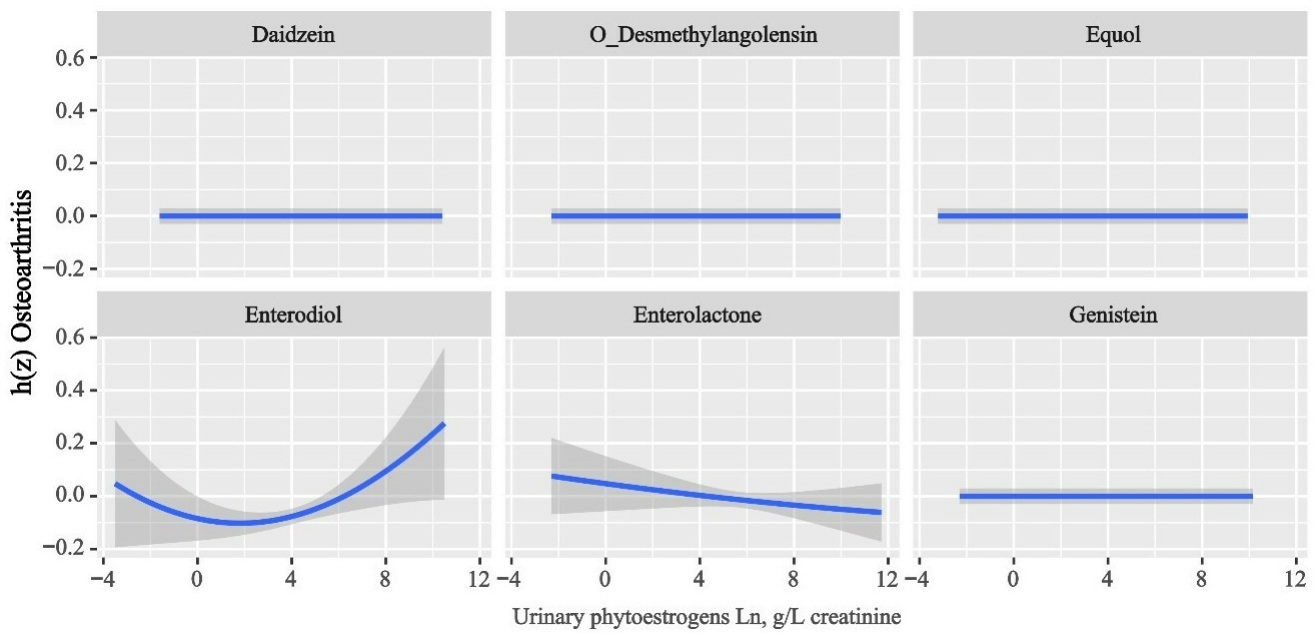
Associations of the urinary phytoestrogens with OA risk estimated by Bayesian Kernel Machine Regression (BKMR). Exposure-response functions for each phytoestrogen with the other phytoestrogens fixed at the median. Models were adjusted for sex, age, race, education, family income-to-poverty ratio, marital status, body mass index, smoking status, drinking alcohol status, and serum cotinine.

Fig 3 displays the exposure-response functions trends for the six urinary phytoestrogen metabolites. It reveals that, when the concentrations of other phytoestrogens were at median levels, there was a positive association between urinary Enterodiol and OA risk, while Enterolactone showed a negative association with OA risk.

**Fig 4:**
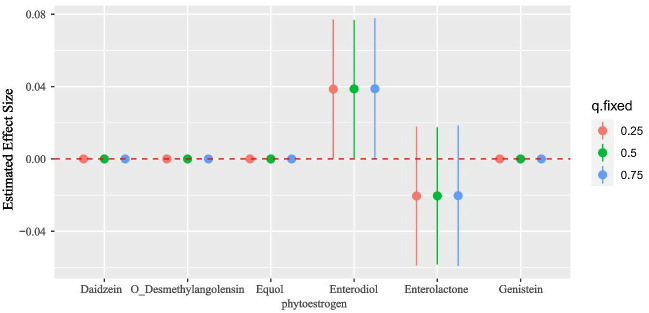
Associations of the urinary phytoestrogens with OA risk estimated by Bayesian Kernel Machine Regression (BKMR). The effect size measures the potential impact of a specific phytoestrogen’s concentration, assessed between the 25th and 75th percentiles. Other phytoestrogens are held constant at their 25th, 50th, or 75th percentiles. An effect size of zero indicates no observed impact. Models were adjusted for sex, age, race, education, family income-to-poverty ratio, marital status, body mass index, smoking status, drinking alcohol status, and serum cotinine.

The concentrations of other phytoestrogens were held constant at the 25th, 50th, and 75th percentiles, and the risk of OA was examined within the range of phytoestrogen concentrations between the 25th and 75th percentiles. The results depicted in Fig 4 reveal a positive relationship between Enterodiol and the risk of OA. Conversely, no statistical association was observed between other phytoestrogens and OA (Fig 4).

**Fig 5:**
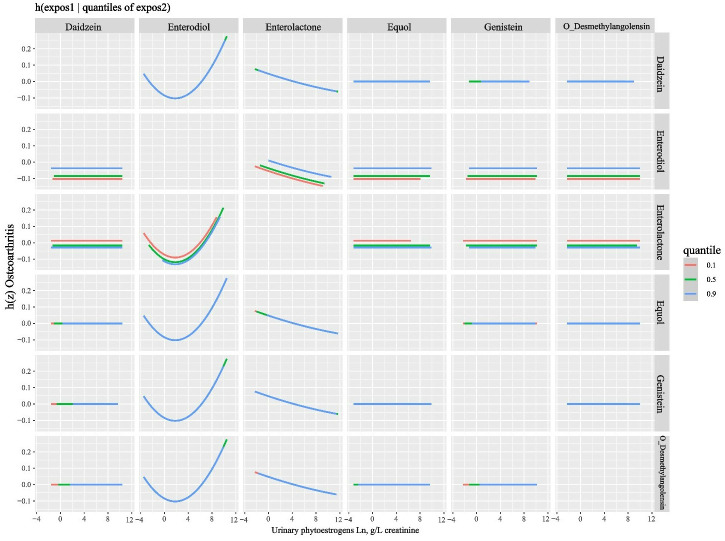
Mean difference in latent continuous variable of osteoarthritis as bivariate exposure-response functions for each of the exposure 1 phytoestrogen when exposure 2 phytoestrogen were at their 10%, 50%, and 90% levels, and other phytoestrogens were fixed at their median levels. In the top-right panel in Fig 5, for example, the exposure-response functions of Daidzein and the latent continuous variable of Osteoarthritis were conducted when O-Desmethylangolensin was fixed at the 10th, 50th, or 90th percentile, and the other four phytoestrogens were fixed at their median levels. Models were adjusted for sex, age, race, education, family income-to-poverty ratio, marital status, body mass index, smoking status, drinking alcohol status, and serum cotinine.

The relationship between the exposure of the first phytoestrogen and OA was examined in relation to the fixed concentration of the second phytoestrogen at the 10th, 50th, and 90th percentile levels, with the other phytoestrogen set at the median level. The results displayed in Fig 5 demonstrate that when the concentrations of the other phytoestrogen were fixed at the median level, the slope of the exposure-response function for one phytoestrogen remained consistent across different quantiles of the other phytoestrogen. This suggests that there is no interaction between any two phytoestrogens in relation to the risk of OA.

## 4 Discussion

This study examines the relationship between urinary phytoestrogen levels and the risk of OA. The data used for analysis was obtained from the 2003–2010 NHANES survey. Weighted logistic regression models were applied, revealing significant associations between enterolactone and enterodiol and the risk of OA. Further analysis using the WQS model indicated a positive relationship between urinary phytoestrogen mixed metabolites and the risk of OA. Specifically, the association between mixed metabolites and OA was primarily driven by enterodiol. When employing the BKMR model, it was observed that the association between urinary phytoestrogen and OA increased with higher phytoestrogen concentrations, although the association did not reach statistical significance. Notably, the univariate exposure-response function showed a positive relationship between enterodiol and OA.

Previous studies have predominantly focused on the relationship between the exposure to a single chemical and the associated health risks, neglecting the concurrent exposure to other chemicals in the statistical analysis. In reality, individuals commonly encounter multiple chemicals simultaneously, exposing them to a multitude of sources. Consequently, novel statistical methods have emerged to evaluate the impact of mixed exposure to multiple chemicals on health outcomes, with the most frequently utilized methods being the WQS regression model and the BKMR model [23]. For instance, a cross-sectional study conducted in the United States employed the WQS model to investigate the relationship between the combined exposure to phenols, phthalates, and polycyclic aromatic hydrocarbons and the risk of frailty. The findings suggested a significant increase in the frailty index with elevated levels of mixed organic pollutants [24]. Through an analysis of the NHANES database, Yang et al. demonstrated a positive relationship between urinary phytoestrogen metabolites mixture and uterine leiomyoma using the WQS model, with Equol exhibiting the highest weight. The BKMR model also indicated a positive association between Equol and the risk of uterine leiomyoma. Chen et al., employing the WQS regression model and BKMR model analysis, found a significant positive relationship between the exposure to mixed urinary metal mixtures and the risk of OA. Importantly, these statistical results were robust and did not notably change after adjusting for confounding variables such as diabetes, hypertension, cardiovascular disease, and medication use for OA [25]. To date, no studies have investigated the relationship between urinary phytoestrogen metabolites mixture and OA.

The results of the analysis using the WQS regression model in this study suggest a positive relationship between urinary phytoestrogen mixed metabolites and the risk of OA. Among these metabolites, Enterodiol was found to have the greatest effect on OA. The BKMR model also supported the association between Enterodiol and increased risk of OA. Enterodiol is a metabolite of plant lignans in the mammalian gut [26], which is catabolized by intestinal flora. Experimental studies have demonstrated that Enterodiol has a biphasic effect on the differentiation of osteoblasts. At low doses, Enterodiol increases cell viability, alkaline phosphatase activity, and the transcription levels of osteonectin and collagen type I in the extracellular matrix. However, at high doses, Enterodiol exhibits an inhibitory effect [27]. Furthermore, an in vitro study has shown that Enterodiol acts as an estrogen receptor agonist and exerts estrogen-like effects [28]. Given that OA is closely associated with human estrogen levels, the estrogen-like effect of Enterodiol may contribute to an increased risk of OA. Currently, the molecular mechanism underlying the relationship between phytoestrogen mixed metabolites and OA remains unclear, and further research is needed to explore the potential mechanism of this association.

This study is subject to several limitations. Firstly, due to the use of data from a cross-sectional survey in the United States (NHANES), it was not possible to establish a causal relationship between urinary phytoestrogen and OA. Secondly, the pathogenesis of OA is multifaceted, and the NHANES database does not include certain potential confounding variables like genetic factors. Thirdly, as the NHANES survey only collected urine samples at a single time point, the concentrations of phytoestrogen metabolites, which may undergo temporal fluctuations [29], could potentially impact the association between phytoestrogen and OA. Consequently, further prospective studies and experiments with larger sample sizes are warranted to explore the link between urinary phytoestrogen and OA, as well as the underlying mechanisms.

## 5 Conclusions

This study utilized three statistical models, namely, the generalized linear model, the WQS model, and the BKMR model, to assess the association between urinary phytoestrogen levels and OA. The findings revealed a significant positive relationship between the collective metabolic composition of urinary phytoestrogens and the risk of OA. Additionally, the presence of Enterodiol emerged as the predominant factor influencing this.

## Supporting information

S1 Table Basic characteristics of participants by OA among U.S. adults, NHANES 2003–2010. (DOCX)

S2 Table OR (95% CI) in OA associated with single phytoestrogens levels. (DOCX)

S3 Table The association between WQS regression index and osteoarthritis. (DOCX)
