# Supplementary material for: Associations between urinary phytoestrogen mixed metabolites and osteoarthritis risk
Source: PLoS One. 2024 Nov 14;19(11):e0313675. doi: 10.1371/journal.pone.0313675 (PMC11563356; doi:10.1371/journal.pone.0313675)
Supplement: S1 Table — (DOCX) [file pone.0313675.s001.docx]

**Table S1 Basic characteristics of participants by OA among U.S. adults, NHANES 2003–2010**

| Characteristics | Total | | Non-OA | OA | *P* value |
| --- | --- | --- | --- | --- | --- |
|  | N=5434 | | N=4841 | N=582 |  |
| Age (years) | 45.40±16.95 | | 43.41±16.11 | 62.34±14.29 | ＜0.001 |
| Sex *n* (%) |  | |  |  | ＜0.001 |
| Male | 2662(49.1) | | 2423(50.2) | 228(39.9) |  |
| Female | 2772(50.9) | | 2418(49.8) | 354(60.1) |  |
| Race *n* (%) |  | |  |  | ＜0.001 |
| Mexican American | 1130(13.7) | | 1072(14.7) | 58(5.6) |  |
| Other Hispanic | 395(5.6) | | 363(5.8) | 32(3.7) |  |
| Non-Hispanic White | 2588(60.6) | | 2186(58.5) | 402(79.0) |  |
| Non-Hispanic Black | 1060(14.4) | | 983(15.1) | 77(9.0) |  |
| Other race | 261(5.6) | | 248(6.0) | 13(2.7) |  |
| Education *n* (%) |  | |  |  | 0.394 |
| Less than high school | 1430(20.5) | | 1278(20.3) | 152(21.8) |  |
| High school or equivalent | 1287(24.0) | | 1158(24.1) | 129(23.0) |  |
| Some college | 1567(30.9) | | 1408(31.2) | 159(28.4) |  |
| College or more | 1150(24.6) | | 1008(24.4) | 142(26.8) |  |
| Marital status *n* (%) |  | |  |  | ＜0.001 |
| Married/cohabiting | 3400(64.1) | | 3053(64.2) | 347(63.4) |  |
| Widowed/divorced/separated | 1067(18.0) | | 869(16.6) | 198(29.5) |  |
| Never married | 967(17.9) | | 930(19.2) | 37(7.0) |  |
| Smoking status |  | |  |  | ＜0.001 |
| Never smoker | 2945(53.7) | | 2688(54.8) | 257(44.3) |  |
| Past smoker | 1268(23.1) | | 1042(21.3) | 226(38.8) |  |
| Current smoker | 1221(23.1) | | 1122(23.9) | 99(17.0) |  |
| Alcohol status |  | |  |  | ＜0.001 |
| Never | | 1507(24.6) | 1284(24.4) | 223(35.0) |  |
| Ever | 3927(75.4) | | 3568(76.6) | 359(65.0) |  |
| BMI (kg/m^2^) | 28.39± 6.37 | | 28.19± 6.31 | 30.03± 657 | ＜0.001 |
| Serum cotinine (ng/mL) | 60.25± 127.89 | | 61.36± 127.92 | 52.32± 130.06 | 0.157 |
| Family PIR | 2.86±1.59 | | 2.85±1.60 | 2.90±1.51 | 0.535 |

Continuous variables were presented as mean ± SE. Categorical variables were presented as *n* (%). *NHANES* National Health and Nutrition Examination Survey, *OA* osteoarthritis, *PIR* the ratio of family income to poverty, *BMI* body mass index, *SE* standard error, *n* numbers of subjects; %, weighted percentage.
