# Supplementary material for: Associations between urinary phytoestrogen mixed metabolites and osteoarthritis risk
Source: PLoS One. 2024 Nov 14;19(11):e0313675. doi: 10.1371/journal.pone.0313675 (PMC11563356; doi:10.1371/journal.pone.0313675)
Supplement: S2 Table — (DOCX) [file pone.0313675.s002.docx]

**Table S2 OR (95% CI) in OA associated with single** **Phytoestrogens levels**

| Characteristics | Quartile 1 | Quartile 2 | *P*  Value | Quartile 3 | *P* Value | Quartile 4 | *P*  Value | Continuous | *P* Value for Trend |
| --- | --- | --- | --- | --- | --- | --- | --- | --- | --- |
|  |  | OR (95% CI) |  | OR (95% CI) |  | OR (95% CI) |  | OR (95% CI) |  |
| Daidzein | Ref | 0.97(0.69,1.34) | 0.857 | 0.95(0.63,1.45) | 0.845 | 1.01(0.72,1.40) | 0.939 | 1.00(0.84,1.19) | 0.967 |
| O-Desmethylangolensin | Ref | 0.78(0.58,1.05) | 0.108 | 1.01(0.72,1.40) | 0.939 | 0.83(0.57,1.22) | 0.365 | 0.97(0.85,1.09) | 0.623 |
| Equol | Ref | 1.15(0.88,1.52) | 0.288 | 1.17(0.88,1.56) | 0.253 | 1.07(0.79,1.45) | 0.644 | 1.01(0.92,1.11) | 0.771 |
| Enterodiol | Ref | 1.06(0.80,1.41) | 0.656 | 1.10(0.82,1.47) | 0.520 | 1.46(1.09,1.96) | 0.010 | 1.14(1.04,1.25) | 0.006 |
| Enterolactone | Ref | 0.89(0.68,1.18) | 0.449 | 0.65(0.48,0.88) | 0.005 | 0.70(0.52,0.96) | 0.027 | 0.86(0.78,0.95) | 0.004 |
| Genistein | Ref | 1.06(0.78,1.45) | 0.684 | 1.05(0.72,1.51) | 0.788 | 1.06(0.68,1.67) | 0.774 | 1.02(0.88,1.18) | 0.777 |

Models were adjusted for sex, age, race, education, family income-to-poverty ratio, marital status, body mass index, drinking alcohol status, smoking status and serum cotinine. Continuous, Ln-transformed concentration of phytoestrogens; Q, quartile; OA, osteoarthritis.
