# Supplementary material for: Associations between urinary phytoestrogen mixed metabolites and osteoarthritis risk
Source: PLoS One. 2024 Nov 14;19(11):e0313675. doi: 10.1371/journal.pone.0313675 (PMC11563356; doi:10.1371/journal.pone.0313675)
Supplement: S3 Table — (DOCX) [file pone.0313675.s003.docx]

**Table S3 The Association between WQS Regression Index and** **Osteoarthritis**

| Outcomes | OR | 95% CI of OR | *P* Value |
| --- | --- | --- | --- |
| Osteoarthritis | 1.18 | (1.02,1.35) | 0.022 |

OR: odds ratio; CI: confidence interval; OR estimates represent the odds ratios of osteoarthritis when the WQS index was increased by one quartile. Models were adjusted for sex, age, race, education, family income-to-poverty ratio, marital status, body mass index, drinking alcohol status, smoking status and serum cotinine.
